# Genomic comparison between cerebrospinal fluid and primary tumor revealed the genetic events associated with brain metastasis in lung adenocarcinoma

**DOI:** 10.1038/s41419-021-04223-4

**Published:** 2021-10-12

**Authors:** Zhiyong Deng, Liang Cui, Pansong Li, Nianjun Ren, Zhe Zhong, Zhi Tang, Lei Wang, Jianwu Gong, Haofeng Cheng, Yanfang Guan, Xin Yi, Xuefeng Xia, Rongrong Zhou, Zhengwen He

**Affiliations:** 1 Department of Neurosurgery, The Affiliated Cancer Hospital of Xiangya School of Medicine, Central South University/Hunan Cancer Hospital, Hunan Province Changsha, 410013 China; 2 GenePlus-Beijing Institute, Beijing, 102206 China; 3 Department of Radiation Oncology, Xiangya Hospital, Central South University, Hunan Changsha, 410008 China

**Keywords:** Cancer genomics, Non-small-cell lung cancer, Oncogenes

## Abstract

Lung adenocarcinoma (LUAD) is most common pathological type of lung cancer. LUAD with brain metastases (BMs) usually have poor prognosis. To identify the potential genetic factors associated with BM, a genomic comparison for BM cerebrospinal fluid (CSF) and primary lung tumor samples obtained from 1082 early- and late-stage LUAD patients was performed. We found that single nucleotide variation (SNV) of *EGFR* was highly enriched in CSF (87% of samples). Compared with the other primary lung tissues, copy number gain of *EGFR* (27%), *CDK4* (11%), *PMS2* (11%), *MET* (10%), *IL7R* (8%), *RICTOR* (7%), *FLT4* (5%), and *FGFR4* (4%), and copy number loss of *CDKN2A* (28%) and *CDKN2B* (18%) were remarkably more frequent in CSF samples. CSF had significantly lower tumor mutation burden (TMB) level but more abundant copy number variant. It was also found that the relationships among co-occurrent and mutually exclusive genes were dynamically changing with LUAD development. Additionally, CSF (97% of samples) harbored more abundant targeted drugs related driver and fusion genes. The signature 15 associated with defective DNA mismatch repair (dMMR) was only identified in the CSF group. Cancer associated pathway analysis further revealed that ErbB (95%) and cell cycle (84%) were unique pathways in CSF samples. The tumor evolution analysis showed that CSF carried significantly fewer clusters, but subclonal proportion of *EGFR* was remarkably increased with tumor progression. Collectively, CSF sequencing showed unique genomic characteristics and the intense copy number instability associated with cell cycle disorder and dMMR might be the crucial genetic factors in BM of LUAD.

## Introduction

Lung adenocarcinoma (LUAD) is the most common histologic subtype in non-small cell lung cancers (NSCLC) and accounts for more than 38.5% of all lung cancers [1]. Brain metastasis (BM) represents an important cause of morbidity and mortality and is associated with poor prognosis [2]. BMs in lung cancer patients (20–56%) are the most commonly arising compared with other tumor types [3]. About 30% of LUAD patients are most likely to suffer from BM at the time of diagnosis, and 50% will eventually develop BMs [4]. The risk of BMs will be increased with increasing tumor grade [5], which has a negative impact on the life quality of patients with LUAD.

Cerebrospinal fluid (CSF), containing cell-free DNA (cfDNA), had been considered as a vital liquid biopsy medium for lung cancer, which provides a less-invasive and routinely accessible method to dynamically acquire genomic information of BM patients in lung cancer [6]. Previous studies had revealed CSF circulating tumor DNA (ctDNA) was more representative of brain tumor genomic alterations than plasma, and could detect brain tumor private mutations and monitor brain tumor progression [7–9]. Besides, CSF cfDNA could enhance the diagnostic validity for *EGFR* genotyping of LUAD patients with BM [10], and reveal frequent occurrence of uncommon *EGFR* mutations (G719A, L861Q, L703P, and G575R) in patients with leptomeningeal metastasis (LM, 54.5%) than brain parenchymal metastasis (BPM, 10%) [11]. Similarly, Ma *et al*. discovered that the mutation ratio of *EGFR* in LM (81.8%) was higher than BPM (30%) in NSCLC patients with BM, and the status of *EGFR* mutation was consistent between CSF ctDNA and brain lesion tissue in five patients after surgical resection [12]. Additionally, CSF DNA genotyping was associated with survival outcomes among LUAD patients with central nervous system (CNS) metastasis [13] and Osimertinib response of LM in EGFR-mutated NSCLC [14]. These studies suggested that CSF could more accurately reflect the genomic mutations of brain lesions in lung cancer patients, thereby providing targets for treatment of BM.

Although amounts of genes in CSF had been identified in previous researches, these studies have a limited the number of samples, and mainly focus on exploring the difference between BM lesions and primary lung tumor in advanced NSCLC, lacking of early-stage genomic comparison. Therefore, a systematic analysis of a large sample size was needed to further explore genetic alterations in the development of lung cancer. In this study, a 1021 cancer-related panel was used to detect gene mutations of 1082 unmatched samples from Chinese LUAD patients, including 135 CSF with BM (CSF group), 363 early-stage lung tumors (ESLT group), 396 late-stage lung tumors without BM (LSLT-noBM group), and 188 late-stage lung tumors with BM (LSLT-BM group) samples. To seek risk factors associated with metastasis by comparing genetic profiles between CSF and different stage primary lung tumors, thereby providing potential prognostic markers and therapeutic targets for LUAD with BMs, finally explaining the development of LUAD from a genetic perspective.

## Results

### Tumor mutation burden and copy number variation instability of the CSF and primary lung tumors

**Fig. 1:**
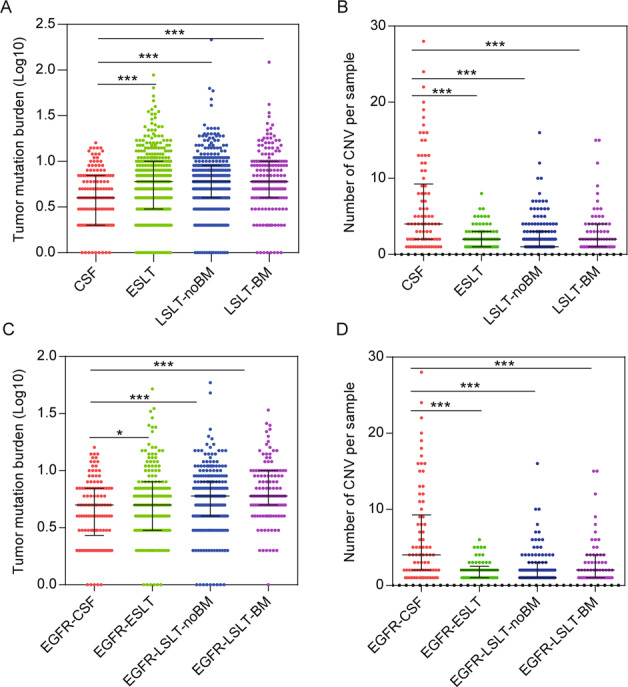
Summary of TMB and CNV count differences across the CSF and other primary lung tissue samples. **A**, **B** Show the difference of TMB and CNV between CSF and ESLT, LSLT-noBM, LSLT-BM groups, respectively. **C**, **D** Show the difference of TMB and CNV in *EGFR*-CSF and *EGFR*-ESLT, *EGFR*-LSLT-noBM, *EGFR*-LSLT-BM subgroups, respectively. Statistical analysis was performed using the Mann–Whitney test. *P < 0.05, ***P < 0.001.

A 1021 panel sequencing was performed on CSF, ESLT, LSLT-noBM, and LSLT-BM groups with the average depth of 1583, 1230, 1271, and 1254×, respectively. Tumor mutation burden (TMB) and somatic copy number variation (CNV) (including amplification and deletion) count were assessed. The median TMB of CSF was 4 mutations/Mb, which was remarkably lower than the other groups (all with a median of 6 mutations/Mb) (Fig. 1A). The median CNV count of CSF was 4, while the median CNV count in ESLT, LSLT-noBM, and LSLT-BM groups were significantly reduced (2, 1, and 2 CNVs, respectively) (Fig. 1B).

We discovered *EGFR* was the most prevalent gene in all groups, accounting for 87% in CSF, 51% in ESLT, 64% in LSLT-noBM, and 67% in the LSLT-BM. Thus, the variation characteristics of *EGFR* mutant patients were further analyzed. The median TMB of *EGFR* mutant CSF (*EGFR*-CSF) and ESLT (*EGFR*-ESLT) samples was 5 mutations/Mb, which was lower than *EGFR*-LSLT-noBM (6 mutations/Mb) and *EGFR*-LSLT-BM samples (6 mutations/Mb) (Fig. 1C). CNV analysis showed that the median count of *EGFR*-CSF subgroup was significantly higher than the other three subgroups (Fig. 1D). These accumulated results suggested that CSF had a lower TMB level but higher CNV instability.

### Single nucleotide variation landscapes and the differences between CSF and other groups

**Fig. 2:**
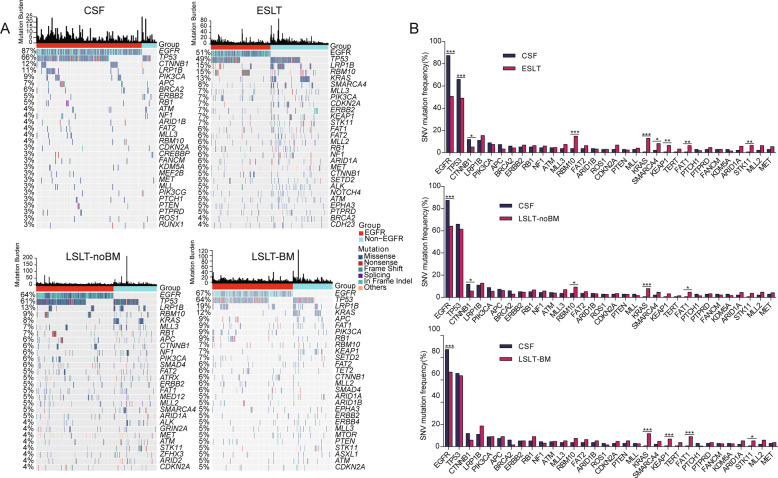
SNVs analysis of LUAD patients at different stages. **A** Driver gene mutation profiles of the CSF, ESLT, LSLT-noBM, and LSLT-BM groups. Mutation frequencies in the group are shown on the left. Mutation burden (number of mutations per Mb) for each patient is shown at the top. **B** Comparison of mutation frequencies of driver genes between CSF and the other three groups, respectively. Significant differences of genes were calculated by two-sided Fisher’s exact test. *P < 0.05, **P < 0.01, ***P < 0.001.

Genomic landscape of single nucleotide variation (SNV) for each group was shown in Fig. 2A. Whereafter, the comparison of mutation prevalence between CSF and the different stages lung tumors revealed that CSF harbored more prevalent *EGFR*, *TP53*, and *CTNNB1*, and fewer *RBM10*, *KRAS*, *SMARCA4*, *KEAP1*, *FAT1*, and *STK11* than ESLT group. The significantly differential genes in CSF and LSLT-noBM groups were *EGFR*, *CTNNB1*, *RBM10*, *KRAS*, and *FAT1*. Gene *EGFR*, *KRAS*, *KEAP1*, *FAT1*, and *STK11* were significantly different between CSF and LSLT-BM group. Moreover, *EGFR* was enriched and *FAT1* was rare in CSF compared to the other three groups (Fig. 2B). Additionally, the incidence of *CTNNB1* and *EGFR* co-mutation in the CSF group was significantly higher than that in the ESLT (11.1% vs 3.6%, *P* = 0.0033), LSLT-noBM (11.1% vs 4.5%, *P* = 0.0117), and LSLT-BM group (11.1% vs 5.3%, *P* = 0.0603), suggesting that the co-mutation of *EGFR* and *CTNNB1* might be associated with BM event.

We also examined the mutation landscape in *EGFR*-mutant patients (Fig. S1A). The results showed that the mutation frequency of *RBM10* was significantly lower in *EGFR*-CSF subgroup than *EGFR*-ESLT subgroup, while the mutation frequency of *TP53* and *PTCH1* in *EGFR*-CSF subgroup was remarkably higher than *EGFR*-ESLT subgroup. *FAT1* was the significantly differential gene between *EGFR*-CSF and *EGFR*-LSLT-BM subgroups, while *EGFR*-CSF and *EGFR*-LSLT-noBM groups had no significantly differential genes (Fig. S1B). *TP53* had an equivalent incidence in patients with advanced tumors, higher than early-stage, indicating the potential association with distant metastasis. In summary, rare gene differences were identified between *EGFR* CSF and other corresponding subgroups.

**Fig. 3:**
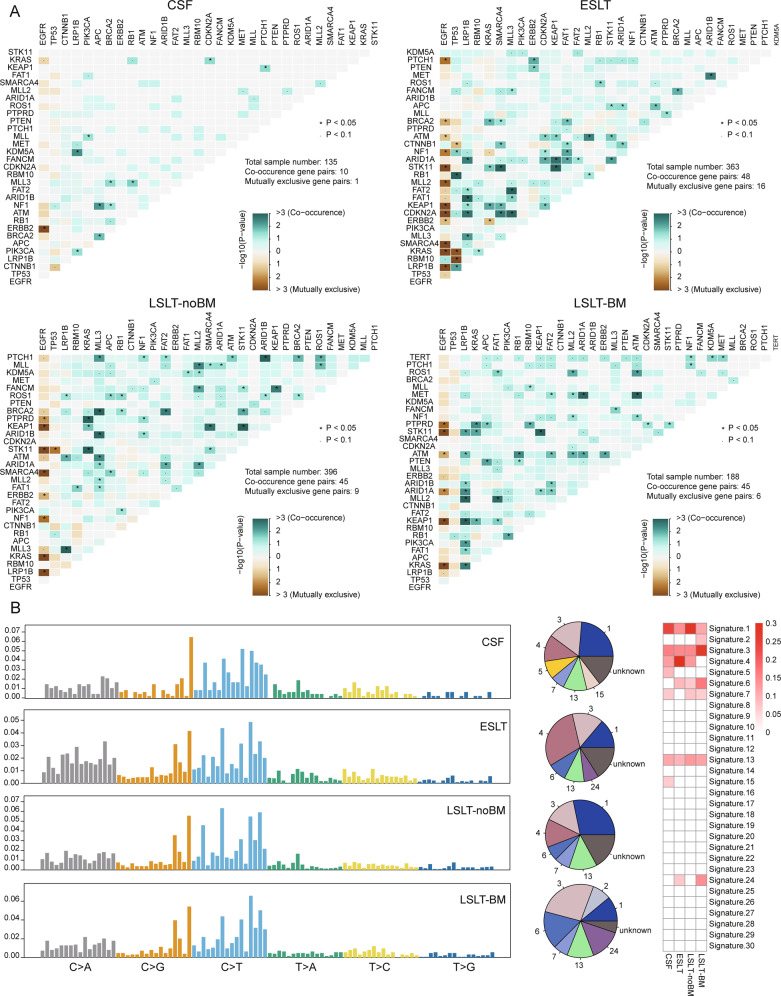
Mutational relationships and processes based on CSF and primary lung tumors. **A** Co-occurrence and mutually exclusivity analyses of somatic mutation genes in CSF and lung tissue. Fisher’s exact test was used to identify remarkable interactions. *P < 0.05. **B** The somatic mutation signature analysis. From left to right: the mutation distribution profile of tumor samples and the vertical axis represents the number of mutations for each triple nucleotide type, proportion of total somatic substitutions in four groups contributed by each of the operative mutational signatures, the heatmap distribution of signatures in all samples.

Co-occurrence and mutually exclusivity analyses among mutant genes showed that *EGFR* and *STK11*/*KEAP1*/*KRAS* were remarkably mutually exclusive genes shared by all lung tissue samples (Fig. 3A). Nevertheless, the gene pairs only co-occurred in CSF group included *LRP1B-KDM5A* (2.2%), *PIK3CA*-*MLL* (1.5%), *APC*-*NF1* (2.2%), *APC*-*BRCA2* (2.2%), *BRCA2*-*NF1* (1.5%), *RB1-MLL3* (1.5%), *CDKN2A*-*KRAS* (0.7%), and *PTCH1*-*KEAP1* (0.7%), reminding their possible relation with BM event. Genes that were specifically mutually exclusive in the ESLT group included *EGFR* and *PTCH1/BRAC2/MLL2/CDKN2A*, *TP53* and *CTNNB1*/*KRAS*/*RBM10*, and *KRAS* and *ERBB2*. The mutual exclusion between *TP53* and *STK11* only appeared in the LSLT-noBM group. The mutually exclusive mutations that only exist in the LSLT-BM group are *EGFR* and *ARID1A*. The above results indicated that the relationships among genes were dynamically changing with LUAD development.

A somatic mutational signature analysis was performed to describe which internal boundary or external environmental factors in the development of LUAD BM represents the most important carcinogenic factors. Signature 1 (correlates with the age of cancer diagnosis), signature 3 (associated with failure of DNA double-strand break repair by homologous recombination), and signature 13 (attributed to the activity of AID/APOBEC family cytidine deaminases) were mutual signatures in both early and late stages LUAD. Notably, signatures 5 and 15 were only identified in the CSF group (Fig. 3B). Although signature 5 has been found in various cancer types, its aetiology is still unknown. Signature 15 is associated with defective DNA mismatch repair (dMMR), an aberrant DNA repair mechanism, contributing to frequent genomic alterations and genomic instability [15], indicating dMMR signature might participate in BM event.

### Somatic CNV landscapes and differences between CSF and the other groups

**Fig. 4:**
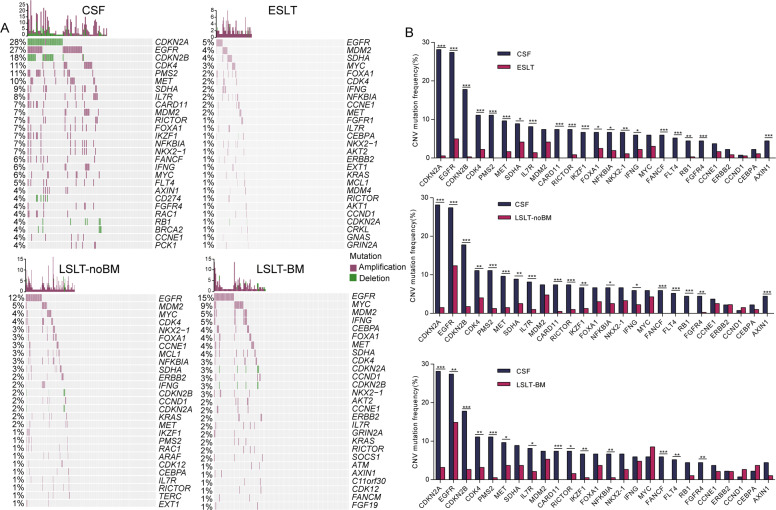
CNVs analysis of LUAD patients at different stages. **A** High frequently mutated genes in CSF and primary lung tumors are shown. Mutation frequencies in the group are shown on the left. CNV counts (number of CNV events) for each patient is shown at the top. **B** Comparison of mutation frequencies of CNV genes between CSF and the other three groups, respectively. Significantly different genes were calculated by two-sided Fisher’s exact test. *P < 0.05, **P < 0.01, ***P < 0.001.

Next, we analyzed CNV features (Fig. 4A, B). Fifty (23.8%) genes were private in CSF group, including amplification of *FANCF* (6%) and *FLT4* (5%) and deletion of *CD274* (4%). Besides, the deletion of *CDKN2A* and *CDKN2B*, and the amplification of *EGFR*, *CDK4*, *PMS2*, *MET*, *IL7R*, *RICTOR*, *FGFR4* were the most frequently observed CNV events in CSF compared with the other groups. Except for the high-frequency genes in CSF mentioned above, the significantly differential genes in CSF and ESLT group also included *SDHA*, *FOXA1*, *NKX2-1*, *IFNG*, *RB1*, and *AXIN1*. The significantly differential genes in CSF and LSLT-noBM group also included *SDHA*, *IFNG*, *RB1*, and *AXIN1*, while the significantly differential genes between CSF and LSLT-BM group were same as high-frequency genes in CSF.

CNVs of *EGFR*-mutated patients also were assessed. The high-frequency genes in the *EGFR*-CSF subgroup and identified differential genes among subgroups also were consistent with the results of total samples (Fig. S2A, B). Collectively, our results demonstrated that CSF derived from BM patients suffered a remarkedly higher level of genetic disturbance events of CNV.

### Brain metastasis related pathways identified using CSF

**Fig. 5:**
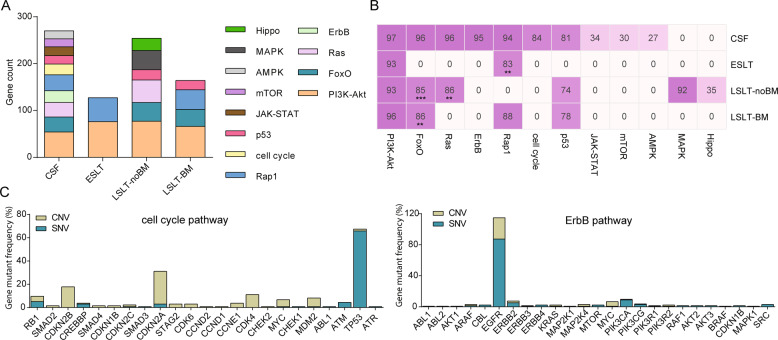
Pathway differences between CSF and primary lesions. **A** Remarkable enrichment of KEGG pathways in each group according to FDR < 0.1. The vertical axis indicates gene count. Gene count means the number of mutated genes enriched in one term. **B** Heatmap of alteration frequency of identified pathways. The horizontal axis represents signal pathways, and the figures indicate mutation percentages. Differences with significant *P* values are labeled (two-sided Fisher’s exact test). **P < 0.01, ***P < 0.001. **C** Gene alterations (including SNV and CNV) of highly frequent and unique pathways in CSF samples. The vertical axis shows gene mutation frequency.

Ten pathways with statistically significant (*FDR* < 0.1) were enriched in CFS, including PI3K-Akt, Rap1, FoxO, Ras, ErbB, cell cycle, JAK-STAT, p53, mTOR, and AMPK pathways. Among them, only ErbB, cell cycle, JAK-STAT, mTOR, and AMPK pathways were remarkably enrolled in the CSF (Fig. 5A), suggesting these aberrant signal pathways might be associated with an increased BM risk. Meanwhile, we performed a significant analysis of alterative frequency in the pathway between CSF and the other three groups (Fig. 5B). The remarkably different pathways in CSF and ESLT groups also included Rap1, CSF vs. LSLT-noBM groups were FoxO and Ras, CSF and LSLT-BM groups were FoxO.

Next, we analyzed gene alterations of ErbB and cell cycle pathways that were highly frequent and unique in CSF samples by integrating SNVs and CNVs. It was found that the somatic mutation of *TP53*, the copy number loss of *CDKN2A* and *CDKN2B*, and the copy number gain of *CDK4* and *MDM2* played major roles in cell cycle pathway, and the somatic mutations of *EGFR* and *PIK3CA* and the amplification of *EGFR*, *ERBB2*, and *MYC* were the main factors affecting ErbB pathway (Fig. 5C).

### Genotyping of the targeted drugs related diver genes

**Fig. 6:**
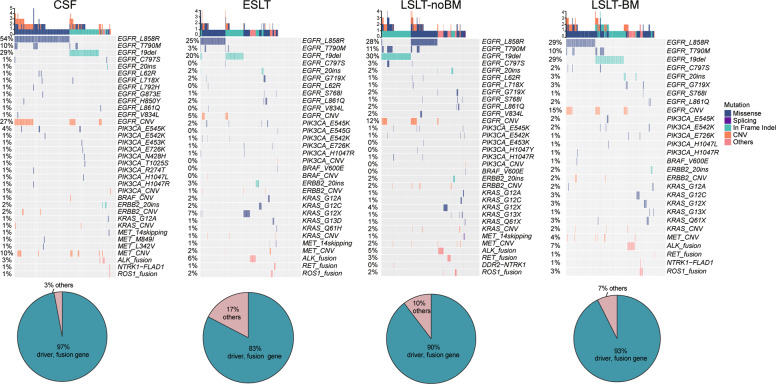
Genotyping profiles of the targeted drugs related diver genes. The mutation landscape of several actionable driver alterations and gene rearrangements in lung cancer is shown at the top. Pie charts at the bottom indicate the proportion of these druggable genes.

We examined the driver genes including *EGFR*, *PIK3CA*, *BRAF*, *ERBB2*, *KRAS*, and *MET*, as well as gene rearrangements such as *ALK*, *RET*, *ROS1*, and *NTRK* fusions. As shown in Fig. 6, these genes accounted for 97% in the CSF group, 83% in the ESLT group, 90% in the LSLT-noBM group, and 93% in the LSLT-BM group. CSF group had significantly higher druggable driver proportion than ESLT (*P* < 0.001) and LSLT-noBM group (*P* = 0.007), excluding LSLT-BM (*P* = 0.091) group. Furthermore, EGFR L858R, T790M, exon 19 deletion (19del), C797S, exon 20 insertion, and L861Q were identified in all groups. Besides, other rare EGFR mutations L62R, L718X, and V834L also were identified in CSF and lung tissue samples, but only L792H, G873E, and H850Y mutated in CSF. *PIK3CA*, *ERBB2*, and *KRAS* were similar in all groups. These results showed that CSF could be used to discover actionable drug-targets in driver genes.

### Clonal architecture of mutations in CSF

**Fig. 7:**
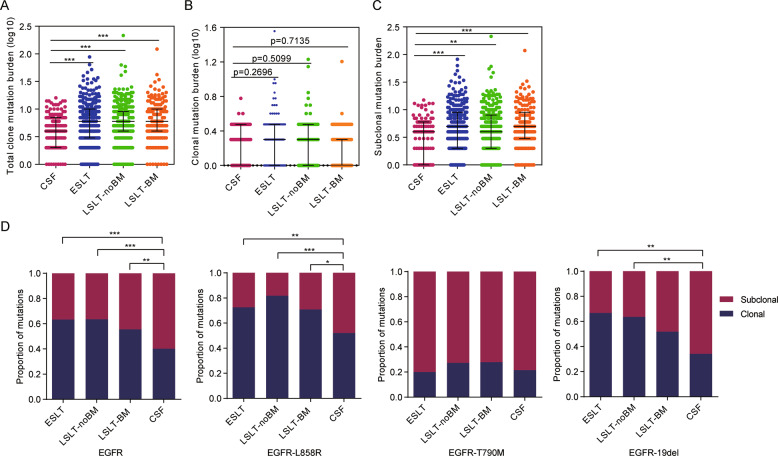
Clonal and subclonal mutations in CSF and primary lung tumors. **A**–**C** Comparison of total clonal mutations burden (including clonal and subclonal), clonal mutation burden, and subclonal mutation burden in all somatic genes for CSF and the other three groups. Clonal mutations burden means the number of mutation clusters in each sample. Differences with significant *P* values are labeled (Mann–Whitney test). **P < 0.01, ***P < 0.001. **D** The clonal and subclonal proportion of *EGFR* and *EGFR* genotyping (L858R, T790M, and 19del) in CSF and primary lung tissue. The differences of these clonal distribution were estimated by two-sided Fisher’s exact test. *P < 0.05, **P < 0.01, ***P < 0.001.

PyClone analysis was performed to explore the mutation clonality in the four groups. We identified 704 mutation clusters in the CSF group with a median of 4 per sample (range, 1–16), 2952 mutation clusters in the ESLT group with a median of 6 per sample (range, 1–88), 3091 mutation clusters in the LSLT-noBM group with a median of 6 per sample (range, 1–215), and 1615 mutation clusters in LSLT-BM group with a median of 6 per sample (range, 1–122). CSF carried significantly fewer clusters than ESLT (*P* = 0.0002), LSLT-noBM (*P* < 0.0001), and LSLT-BM (*P* < 0.0001) group (Fig. 7A). Further analysis revealed that the difference in total clonal mutation burden was mainly due to more subclonal mutations rather than clonal mutations (Fig. 7B, C).

Clonality of the high-frequency genes among groups was further analyzed, in which the subclonal proportion of *EGFR* was increased in CSF. Further, the subclonal fraction of EGFR L858R and 19del in CSF was significantly higher than the other three groups, while subclonal EGFR T790M had no difference (Fig. 7D), suggesting the changes in the distribution of the main genotyping subclones might be one of the reasons for the increase in *EGFR* subclones. Besides, the samples with subclonal EGFR L858R and 19del were characterized by the frequently clonal *TP53*.

## Discussion

In the current study, we analyzed genomic profile difference of Chinese LUAD patients between CSF samples and other primary lung tissues from different disease stages, and proved the unique molecular characteristics of CSF samples from multiple aspects, such as SNV, CNV, signature, clonality and pathway, and revealed CSF cfDNA was an important medium to expound the molecular features of BM LUAD patients, which provided potential prognostic markers and therapeutic targets for LUAD with BMs.

SNVs analysis showed that *EGFR* had a significantly higher mutation frequency in CSF, followed by the LSLT-BM, LSLT-noBM, and ESLT group, which might be contributed by a higher incidence of BMs for patients with *EGFR* mutation NSCLC [16]. Furthermore, mutation frequency of *KRAS* in the CSF group only was 0.7%, which was the lowest in all samples. This was owing to the mutual exclusivity of oncogenic *KRAS* and *EGFR* mutations in LUAD [17, 18]. Herein, *CTNNB1* was highly mutated in CSF. According to reports, *CTNNB1* belongs to Wnt signal pathway and is mutated in patients of lung metastasis to brain, but few or no mutations were described in original brain tumor specimens [19]. Combining with our data, *CTNNB1* was indeed required for lung cancer with BM. Another study found that *CTNNB1* mutations were rare in early-stage *EGFR*-mutant LUAD, but increased in late-stage tumors [20]. In our results, the number of *EGFR* and *CTNNB1* co-mutation in CSF samples was significantly more than that in ESLT and LSLT-noBM lung tissue samples, but not LSLT-BM samples, indicating that the more overlap of *EGFR* and *CTNNB1* was related to BM in LUAD.

Somatic mutations and copy number alterations possessed intratumor heterogeneity, and genome doubling and continuous dynamic chromosomal instability were related to intratumor heterogeneity, leading to parallel evolution of driver somatic copy-number alterations [21]. In our study, more abundant CNVs were observed in CSF cfDNA, suggesting that CSF had stronger genome instability, which was in accordance with the hypothesis that large-scale genomic alterations, such as copy number changes, is often evident at metastatic sites [22], and genome instability drives tumor progression and metastasis [23]. On contrast, minimum TMB was found in CSF. This was owing to highly frequent *EGFR* in CSF, which had been verified by a previous study that lung cancer patients with *EGFR* mutations had significantly lower TMB values than those with wild-type *EGFR* [24]. Also, the difference of somatic mutation genes among groups much less than that of copy number alteration genes, thus we considered that CNVs of CSF might play more important roles in the evolution of LUAD compared with SNVs. Besides, the frequency of NSCLC gene alterations in CSF was much higher than other groups, including *CDKN2A*/*2B* deletions, and *PMS2*, *MET*, and *CDK4* amplification. Among these observations, it is known that *CDKN2A*/*2B* and *CDK4*, which are involved in cell cycle pathway, were high frequency mutated genes in BM tissue samples, and indicated shortened intracranial progression-free survival in CSF [4, 14, 25]. *PMS2* is related to DNA mismatch repair and might accelerate tumorigenesis [26], and *MET* copy number gains are associated with EGFR tyrosine kinase inhibitors (TKIs) resistance in NSCLC with BMs [27]. Moreover, the amplification of *FANCF* and *FLT4* and the deletion of *CD274* only were identified in CSF samples. Studies had shown that the overexpression of *FANCF* and *FLT4* resulted in proliferation, migration, and invasion of cancer cells [28, 29]. Deletion of *CD274* was most prevalent and frequent in NSCLC, which influenced its expression levels, was associated with dismal prognosis [30]. In general, the above CNVs genes might act in promoting LUAD BMs.

Somatic mutations found that cancer genomes may be the consequence of the intrinsic slight infidelity of the DNA replication machinery, exogenous or endogenous mutagen exposures, enzymatic modification of DNA, or defective DNA repair [31]. Furthermore, the molecular mechanisms underlying genomic instability are related to processes that preserve genetic information, namely cell cycle checkpoints, DNA repair, transcription, replication, epigenetic control, chromatin remodeling, and chromosome segregation during mitosis [15]. Most of the somatic mutations in tumors are induced by exposure, and various predisposing factors have different characteristics in the mutation profile. Different mutational processes often generate different combinations of mutation types, termed “signatures” [32]. In this study, signature 5 and 15 only were detected in CSF, in which signature 5 primarily features C > T and T > C transitions, but its proposed aetiology is currently unknown [33]. A study found that signature 5 is associated with DNA repair gene *ERCC2* mutations in urothelial tumors [34], which may be used for reference to lung cancer research. Signature 15 is associated with dMMR, resulting in genomic instability. Additionally, cell cycle and ErbB pathways also only were frequently enriched in CSF. cell cycle was the one of molecular mechanism contributing to genomic instability as mentioned earlier. As a key pathway of angiogenesis, the ErbB signaling pathway has been implicated in the development of NSCLC patients with BMs as evidenced by overexpression and higher activity than in corresponding primary tumors [35]. Collectively, we suggested that CSF did have much higher genomic instability. dMMR, the alterations of cell cycle and ErbB pathways might be the reasons to induce LUAD BM.

At present, several studies had confirmed that *EGFR* targeted therapy dramatically improved the prognosis of patients with NSCLC BMs [36, 37]. However, concomitant genetic alterations (such as *CDKN2A* copy number loss) [38] and the number of *EGFR* subclones [39] are associated with worse clinical outcomes in *EGFR* mutant NSCLC patients under TKIs treatment. In our study, *CDKN2A* loss and *EGFR* subclone number were frequently increased in *EGFR*-CSF patients, which might be one of the reasons for the poor prognosis of LUAD patients with BM. In immune checkpoint inhibitors (ICIs) monotherapy, *EGFR* mutation patients did not respond better than chemotherapy [40, 41], and *EGFR* aberrations had been considered as a risk factor for hyper-progress in immunotherapy [42]. ICI efficacy against NSCLC patients with *MET* amplification seemed close to that observed in unselected patients [43]. Here, *EGFR* and *MET* generally occurred in CSF samples, suggesting patients with BMs might be more not suitable for immune monotherapy.

Genomic comparison of CSF and primary lung lesions at different stages also revealed several other possible therapeutical approaches for LUAD with BM. CDK4/CDK6 inhibitor abemaciclib had been verified to enhance radiosensitivity of NSCLC in vitro and in vivo [44]. *CDK4* was remarkably amplified in CSF, supporting that the CDK4 inhibitors combined with radiation might be a promising therapeutic option for LUAD patients with BM. ICIs against programmed death-1 (PD-1) and cytotoxic T lymphocyte antigen-4 (CTLA-4) proteins were highly effective in advanced dMMR metastatic colorectal cancer, especially combination therapy provided improved efficacy relative to anti-PD-1 monotherapy [45, 46]. We discovered dMMR mutational signature in CSF of LUAD patients. This provided a potential target for ICI combination therapy. In addition, it has recently been reported that the infiltrating immune cells in CSF could predict the response of BM patients to ICI [47], and diver gene mutations were associated with ICI response [48]. Thus, exploring the effect of special gene alterations in CSF on immune cells might provide guidance for screening patients who benefit from immunotherapy for lung cancer brain metastasis.

To conclude, a multiple perspective analysis about molecular characteristics between CSF and different stages primary tumor tissues in LUAD patients revealed that CNV might be greater than the contribution of SNV to BM. ErbB pathway alterations, and the intense copy number instability associated with cell cycle disorder and dMMR might be the crucial genetic factors in BM of LUAD.

## Materials and methods

### Patients and sample collection

Genomic and clinical information of 1082 LUAD patients with or without BM were analyzed, including 135 (12%) IV stage CSF with BM patients (CSF group), 363 (34%) I–III stage lung tissue patients (ESLT group), 396 (37%) IV stage lung tissue without BM patients (LSLT-noBM group), and 188 (17%) IV stage lung tissue with BM patients (LSLT-BM group). The clinical characteristics and mutated *EGFR* prevalence of patients were summarized in Table 1. Ten milliliter CSF was obtained by lumbar puncture. Surgical tumors fixed in formalin and embedded in paraffin (FFPE) samples were collected for DNA extraction. Peripheral blood lymphocytes (PBLs) from each patient were collected for germline DNA sequencing. All patients provided informed consent. The present study was approved by Hunan Cancer Hospital IRB Committee.

**Table 1:** Characteristics of samples between different groups in this study.

Charactistics	CSF group	ESLT group	LSLT-noBM group	LSLT-BM group
	(*n* = 135)	(*n* = 363)	(*n* = 396)	(*n* = 188)
Stage at initial diagnosis (I-IV)	IV	I–III	IV	IV
Brain metastases (Yes or No)	Yes	No	No	Yes
Median age (range), yrs	53 (18–76)	62 (28–94)	60 (26–85)	58.5 (29–80)
Gender, No. (%)				
Male	66 (49%)	204 (56%)	200 (51%)	98 (52%)
Female	69 (51%)	159 (44%)	196 (49%)	90 (48%)
Histology, No (%)				
Adenocarcinoma	135 (100%)	363 (100%)	396 (100%)	188 (100%)
Smoking history, No. (%)				
Smoker	25 (19%)	98 (27%)	103 (26%)	57 (30%)
Non-smoker	56 (41%)	144 (40%)	165 (42%)	62 (33%)
Unknown	54 (40%)	121 (33%)	128 (32%)	69 (37%)
*EGFR* mutation patient, No (%)	118 (87%)	184 (51%)	254 (64%)	126 (67%)

### DNA extraction

CSF cfDNA was isolated using MagMAX^TM^ Cell-Free DNA Isolation Kit (Thermo Fisher Scientific, Waltham, MA, USA). Lung tumor tissue DNA was extracted using Maxwell® 16 FFPE Plus LEV DNA Purification Kit (Promega, Madison, WI, USA). PBL DNA was extracted using the DNeasy Blood & Tissue Kit (Qiagen, Hilden, Germany). DNA concentration was measured using a Qubit fluorometer and the Qubit dsDNA HS (High Sensitivity) Assay Kit (Invitrogen, Carlsbad, CA, USA).

### Library construction and panel sequencing

Methods for sequencing libraries was previously described [49]. DNA extracted from PBL and FFPE specimen sheared to 300-bp fragments with a Covaris S2 ultrasonicator (Covaris, Woburn, MA, USA). CSF DNA and fragmented PBL and tissue DNA (1.0 μg input) was added to Illumina-indexed adapters for library construction using the KAPA Library Preparation Kit (Kapa Biosystems, Wilmington, MA, USA). Custom-designed probes, which covered 1.0 Mb regions for 1021 cancer-related genes were used for DNA capture [50]. Sequencing was performed on the HiSeq3000 Sequencing System (llumina, USA) with 2 × 75 bp paired-end reads.

Sequence analysis used BWA [51] (version 0.7.12-18r1039) to align clean reads to the reference human genome (hg19). SNVs and small Indels were identified by MuTect (version 1.1.4) [52]. Somatic mutations were identified by a variant allele fraction (VAF) ≥ 1.0% and at least 5 high quality reads (Phred score ≥30, mapping quality ≥30, and without paired-end reads bias). Mutations were annotated with genes using the ANNOVAR software [53]. CONTRA was used to detect CNV [54]. BreakDancer was used to detect cancer-associated gene fusion [55].

### TMB and CNV count analyses

CSF-based and tissue-based TMB were defined as the total number of non-synonymous SNVs and Indels standardized by the 1.0 Mb coding region. CNV count was defined as the total number of CNV events per sample.

### Subclonal analysis

Pyclone, a Bayesian clustering method, was employed to estimate the subclonal architecture of all mutations from CSF and primary tumor tissues. The SNV of each sample and its copy number information are used as the input of PyClone analysis, and the cellular prevalence was inferred and variants were clustered as previously described [56]. PyClone was run with 20,000 iterations and default parameters. Variants located in the cluster with greatest cancer cellular prevalence (CCF) mean were defined as clonal, the rest were subclonal.

### Signature analysis

DeconstructSigs package (version 1.8.0) was used to identify mutational signatures within a single tumor sample based on a negative matrix factorization (NMF) algorithm [57], which relies on the Bioconductor library BSgenome.Hsapiens.UCSC.hg19 to obtain mutational context information. The unique combination of mutation types in CSF and lung tissue samples were constructed, and the mutational process was generated by COSMIC mutational signatures (version 2.0).

### Pathway analysis

An online analysis tool DAVID (https://david.ncifcrf.gov/tools.jsp) was performed to identify significant gene clusters from SNVs and CNVs via annotating KEGG pathways, and 1021 cancer-related genes was considered as background gene set. The mutational frequency of pathways was obtained by computing the fraction of samples with at least one alteration in the corresponding pathway [58].

### Statistical analysis

Data were analyzed using Prism 6.0 (Graph Pad Software Inc., La Jolla, CA). The Fisher’s exact test was used to compare proportions between two groups. Mann–Whitney test was used for CNV count of per sample and TMB comparison among different groups. All statistical tests were two-sided, and the result with *P* < 0.05 was considered as statistically significant.

## Supplementary information

FigS1

FigS2

Supplementary figure legends

## Data Availability

The datasets supporting the conclusions of this article are available from the corresponding author on reasonable request.
