# Supplementary material for: Genomic comparison between cerebrospinal fluid and primary tumor revealed the genetic events associated with brain metastasis in lung adenocarcinoma
Source: Cell Death Dis. 2021 Oct 12;12(10):935. doi: 10.1038/s41419-021-04223-4 (PMC8511004; doi:10.1038/s41419-021-04223-4)
Supplement: Supplementary file 3 — Supplementary figure legends [file 41419_2021_4223_MOESM3_ESM.docx]

**Supplementary Figure Legends**

**Fig. S1 SNV profiles in *EGFR*-mutant patients.**

A. SNV landscape of the *EGFR*-CSF, *EGFR*-ESLT, *EGFR*-LSLT-noBM and *EGFR*-LSLT-BM samples. Mutant frequencies in the subgroup are shown on the left. Mutation burden (number of mutations per Mb) for each patient is shown at the top.

B. Comparison of mutation frequencies of driver genes between *EGFR*-CSF and the other three subgroups, respectively. Significant differences of genes were calculated by two-sided Fisher’s exact test. **P* < 0.05, ****P* < 0.001.

**Fig. S2 CNVs analysis in *EGFR*-mutant patients.**

A. High frequently mutated genes in *EGFR*-CSF and primary lung tumors are shown. Mutant frequencies in the subgroup are shown on the left. CNV counts (number of CNV events) for each patient is shown at the top.

B. Comparison of mutation frequencies of CNV genes between *EGFR*-CSF and the other three subgroups, respectively. Significantly different genes were calculated by two-sided Fisher’s exact test. **P* < 0.05, ***P* < 0.01, ****P* < 0.001.
